# Obesity-Mediated Immune Modulation: One Step Forward, (Th)2 Steps Back

**DOI:** 10.3389/fimmu.2022.932893

**Published:** 2022-06-30

**Authors:** Viviane Schmidt, Andrew E. Hogan, Padraic G. Fallon, Christian Schwartz

**Affiliations:** ^1^Mikrobiologisches Institut - Klinische Mikrobiologie, Immunologie und Hygiene, Universitätsklinikum Erlangen and Friedrich-Alexander-Universität (FAU) Erlangen-Nürnberg, Erlangen, Germany; ^2^Kathleen Lonsdale Human Health Institute, Maynooth University, Maynooth, Ireland; ^3^Obesity Immunology Research, St. Vincent’s University Hospital and University College Dublin, Dublin, Ireland; ^4^Trinity Biomedical Sciences Institute, School of Medicine, Trinity College Dublin, Dublin, Ireland; ^5^Medical Immunology Campus Erlangen, Friedrich-Alexander-Universität (FAU) Erlangen-Nürnberg, Erlangen, Germany

**Keywords:** obesity, Th2 (type-2) immune responses, T helper cell 2, metabolism, helminth, malnutrition, adipokine cytokines

## Abstract

Over the past decades, the relationship between the immune system and metabolism has become a major research focus. In this arena of immunometabolism the capacity of adipose tissue to secrete immunomodulatory molecules, including adipokines, within the underlying low-grade inflammation during obesity brought attention to the impact obesity has on the immune system. Adipokines, such as leptin and adiponectin, influence T cell differentiation into different T helper subsets and their activation during immune responses. Furthermore, within the cellular milieu of adipose tissue nutrient availability regulates differentiation and activation of T cells and changes in cellular metabolic pathways. Upon activation, T cells shift from oxidative phosphorylation to oxidative glycolysis, while the differential signaling of the kinase mammalian target of rapamycin (mTOR) and the nuclear receptor PPARγ, amongst others, drive the subsequent T cell differentiation. While the mechanisms leading to a shift from the typical type 2-dominated milieu in lean people to a Th1-biased pro-inflammatory environment during obesity are the subject of extensive research, insights on its impact on peripheral Th2-dominated immune responses become more evident. In this review, we will summarize recent findings of how Th2 cells are metabolically regulated during obesity and malnutrition, and how these states affect local and systemic Th2-biased immune responses.

## Introduction

Obesity has become a major health problem, especially in first world countries, affecting about one third of the population worldwide (1). Individuals with a body-mass-index (BMI) >30 kg/m^2^ are classified as obese, and develop an immune response that, as part of the metabolic syndrome, increases the risk of non-communicable diseases such as type 2 diabetes, hypertension, cardiovascular disease, asthma and cancer (2). Reasons for the development of obesity are multifactorial. By far the most important factor is a chronically dysregulated energy balance – with more energy being taken up than being burnt and thus stored as triglycerides in adipocytes. This leads to the accumulation of triglycerides in adipocytes, increasing the fat mass, which ultimately causes decreased blood flow and oxygen availability in the adipose tissue, cell death, and mechanical stress on the connective tissue. Together with the increased permeability of the gut and disseminated bacterial products, a low grade chronic inflammatory response develops, which leads to local and peripheral dysregulation of T cell polarization.

The impact of the inflammatory state that obesity elicits throughout the body was recently highlighted by the greater severity and poorer outcome following infection with severe acute respiratory syndrome coronavirus 2 (SARS−CoV−2) in individuals with obesity relative to lean counterparts (3). In the past decade, due to the increasing recognition of obesity and the resulting altered metabolism, research has increasingly focused on the influence of the individual’s metabolism as well as cellular metabolism on the immune system and vice versa.

Cellular metabolism is a complex, dynamic process of consuming available nutrients in the cellular environment and producing new metabolites ensuring the proper function of the cell. Depending on the activation status of a cell, their metabolic requirements change. Quiescent cells generate energy using mitochondrial pathways, such as fatty acid oxidation or the tricarboxylic acid (TCA) cycle. These pathways are highly efficient in adenosine triphosphate (ATP) generation and allow for a constant energy production in long-lived cells. Upon cell activation, metabolic reprogramming occurs to adjust to the metabolic requirements. Immune responses evolved to be energetically costly even affecting maintenance programs, such as homeothermy, resulting in physiologic trade-offs (4). Activated cells rapidly produce high amounts of ATP using glycolytic pathways to energize the differentiation and proliferation of cells. With a production of two mole ATP per unit glucose, glycolysis is less efficient in energy production in comparison to mitochondrial catabolic pathways (5). However, this inefficiency is offset as glycolysis can be upregulated faster than mitochondrial metabolic pathways since it does not require mitochondrial growth. Additionally, glycolysis provides biosynthetic intermediates that can further be utilized for ribose synthesis and other essential pathways and therefore represents a dominant metabolic pathway in activated cells (6). Naïve immune cells in the steady state are characterized by a quiescent metabolic profile. Antigen contact or stimulating signals, such as inflammatory cytokines, lead to the activation and subsequent metabolic reprogramming of these cells. For example, type 1 inflammatory macrophages are characterized by energy production *via* glycolytic pathways, while the anti-inflammatory M2 macrophages (M2) predominantly utilize fatty acid oxidation and oxidative phosphorylation. For fatty acid oxidation, fatty acids are first activated to fatty acid acyl-CoA in the cytosol, and then degraded *via* β-oxidation in the mitochondria producing acyl-CoA, NADH and FADH_2_ that can then be used for ATP generation (5). Additionally, it was shown that inhibition of fatty acid oxidation promotes antimicrobial macrophage functions (7).

Cellular functions and metabolism are not only dependent on cell-intrinsic factors, such as activation status, but also on their local environment as well. A change in the composition or quantity of nutrients available within a cellular milieu can lead to differences in the type and magnitude of cellular pathways activated and thereby consequently changes the cell’s function (8). Therefore, changes in the metabolic status of an organism, such as the development of obesity or underweight due to over- or undernutrition, respectively, can influence the metabolism of single cells. An oversupply of nutrients leads to the expansion of adipose tissue (AT) and the progression to overweight and obesity. Based on their distinct functions, AT can be divided into brown (BAT) and white (WAT) adipose tissue, as reviewed by Frigolet et al. (9). BAT is a thermogenic tissue characterized by a high number of mitochondria, while WAT functions as lipid storage. In recent years, adipose tissue has gained a new reputation beyond the previous view as a site of fat deposition for energy storage. AT is now considered a secondary immune organ, with an abundance of leukocytes that populate the tissue, which may act as a reservoir of immune cells and mediators during immune responses. Indeed, novel single-cell sequencing data of cells present in human and mouse adipose tissue underpin the differences of immune cells between the AT of lean and obese individuals (10, 11). These findings led to the conclusion that the immune system and the metabolic state of the organism are closely linked and regulate each other – making the AT one of the most important immunometabolic modulators within the body.

This dynamics of the prevailing immune cell repertoires within the AT is exemplified by the marked differences in the cell composition of AT in persons with or without obesity. In individuals with a lean bodyweight, populations of anti-inflammatory cells such as regulatory T cells (Treg), T helper (Th) type 2 cells, group 2 innate lymphoid cells (ILC2), M2, and eosinophils dominate the AT milieu and may serve to counteract inflammation (12). Th2 cells are classically regarded as important effector cells that produce the hallmark cytokines IL-4 and IL-13, but also IL-3 and IL-5 that promote basophilia and eosinophilia, respectively. Following activation *via* the TCR and co-stimulation, in the presence of type 2 polarizing cytokines, naïve T cells differentiate into Th2 cells expressing the master transcription factor GATA3. These type 2 polarizing factors include the presence of IL-4, which activates the STAT6-signaling pathway; activation of STAT5-signaling through cytokines, such as IL-2, IL-7 and TSLP; weak TCR signaling strength; certain costimulatory molecules and activation of Jag1/Notch-interaction (reviewed in (13)). Downstream effects of Th2 cell activation and release of type 2 cytokines include the differentiation of M2 macrophages, basophilia, eosinophilia, B cell antibody isotype class-switching to IgG1 and IgE. This spectrum of type 2 responses is associated primarily with parasitic infections and allergies. Th2 responses also inhibit Th1 responses and vice versa. During obesity on the other hand, infiltration and/or expansion of pro-inflammatory immune cells, such as inflammatory macrophages, cytotoxic CD8^+^ T cells and Th1 cells in white adipose tissue leads to a constant low-grade inflammation and consequently to metabolic dysfunctions underpinning the obesity-related metabolic syndrome. Mucosal associated invariant T (MAIT) cells are another subset of T cells implicated in obesity related dysfunction (14–19). In people with obesity, MAIT cell frequencies are reduced and biased towards a Th17 phenotype and can directly disrupt metabolic processes such as insulin-mediated glucose uptake (14, 16). Their contribution to obesity-related metabolic dysfunction is further supported by data from murine models, where MAIT cell deficiency protects against metabolic dysfunction (17). MAIT cells from persons with obesity (PWO) display an exhausted phenotype (elevated PD-1) and elevated rates of apoptosis (14, 15). Interestingly, chronically stimulated MAIT cells increase their expression of GATA3 and production of Th2 cytokines such as IL-13 (20). But whether chronic activation in obesity drives Th2 like MAIT cells has not been investigated to date.

Moreover, obesity, and indeed malnutrition, not only affect adipose tissue inflammation but extend to peripheral tissues, where they interfere with systemic immune cell activation and functions. While obesity is often associated with a metabolically diseased state, metabolically healthy PWO constitute up to 50% of PWO (21). However, only few studies have addressed the differences in metabolically healthy and unhealthy PWO with regard to immune cell function. Generally, metabolically healthy PWO still harbor less pro-inflammatory cells within the AT, including M1 macrophages as well as Th17 and Th22 cells (22–24), while Th2 cells correlated with insulin sensitivity (25). The mechanisms causing progression from a metabolically healthy obese state to metabolic disease are still under investigation. Remarkably, mitochondria from type 2 diabetes patients appear altered in their function to promote Th17 cytokine production (26). Although much progress has been made over the past decade (27, 28), further studies stratifying metabolically healthy and diseased PWO are still required to elucidate the underlying processes.

The mutual influence of the immune system and metabolism is an important topic and immunometabolism is an arena of current active research. Here, we will review the current understanding of the metabolic regulation of T helper cells and, in particular, Th2 cells in obesity in contrast to expansion of pro-inflammatory Th subsets. Furthermore, we will summarize recent findings on the dysregulation of Th2-biased immune responses during obesity and malnutrition.

## T Cell Metabolism

T cell metabolism is, as in other immune cells, dependent on the activation status of the cell. As quiescent cells with little need for the *de novo* synthesis of DNA, proteins and lipids, naïve T cells as well as memory T cells produce energy using oxidative phosphorylation (OXPHOS). Effector T cells, on the other hand, switch from mitochondrial pathways to glycolysis due to the rapidly increasing energy consumption following activation. After T cell receptor (TCR) stimulation, expression of genes involved in glycolysis and glutaminolysis are upregulated (29–31). Initially, glycolysis is the dominant metabolic pathway in effector T cells. Transcription factors including c-myc and the hypoxia inducible factor 1 alpha (HIF-1α) control upregulation of glucose. Recently, it was shown that the switch from the quiescent state in CD4^+^ T cells is mediated intracellularly *via* Akt and STAT5 signaling that increased both glycolysis and OXPHOS (32). Upon CD28 ligation, expression of the glucose transporter (Glut) 1 and glucose uptake of CD4^+^ and CD8^+^ effector T cells are increased *via* the PI3K-AKT axis (8, 33, 34). Downstream of PI3K, the kinases mTOR and AMPK as well as the nuclear hormone receptor PPARγ regulate the cell metabolism and differentially influence T cell differentiation. Importantly, Glut1 is essential for CD4^+^ but not CD8^+^ T cells (35). Without the co-stimulatory signal received through CD28-signaling, T cells enter the anergic state (8). Following activation of tumor necrosis factor receptor-associated factor 6 (TRAF6), effector T cells again start utilizing fatty acid oxidation over glycolysis for energy generation and develop into memory T cells (36).

In essence, glycolysis primarily supports the rapid generation of Th1 and Th17 inflammation and promotes IL-2 and IFNγ production. While Th2 cells initially utilize glycolysis, lipid metabolism pathways, such as fatty acid oxidation, synthesis and uptake are upregulated and play an important role in late activation and tissue adaptation (37). Similar to Th2 cells, Tregs mainly generate energy using OXPHOS (38). Importantly, studies investigating differentiation into effector, memory or regulatory T cells, often focused on the use of etomoxir, a drug supposed to inhibit the central enzyme responsible for limitation of long chain fatty acid oxidation, Cpt1a (39–41). However, Raud and colleagues showed that Cpt1a is largely dispensable in this context, implying that etomoxir may exert its functions by alternative mechanisms involving mitochondrial respiration (42).

**Figure 1:**
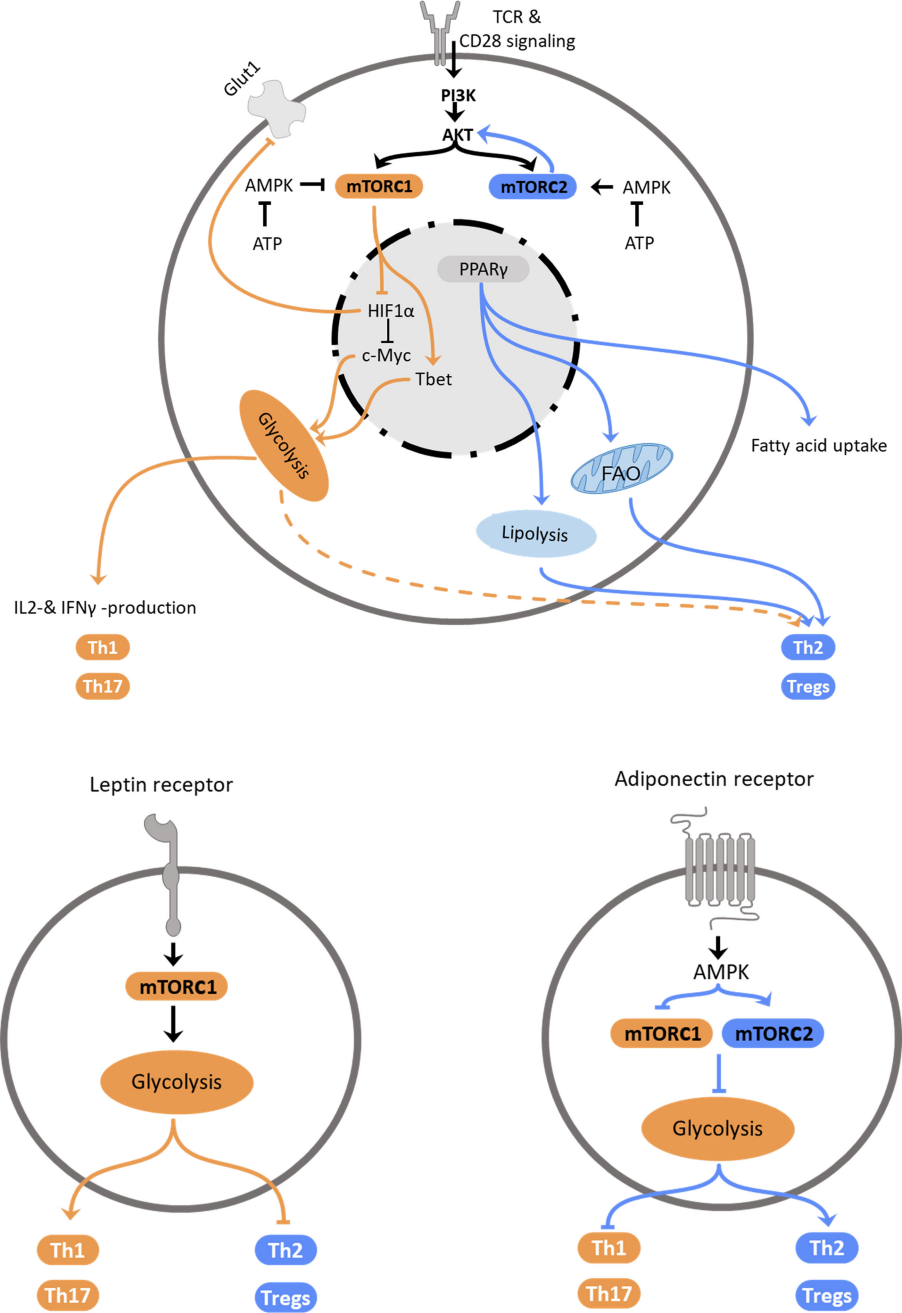
Regulation of Th2 metabolism. Top: Schematic representation of intracellular mechanisms affecting T helper cell polarization. Central elements influencing cellular polarization include mTORC1 and mTORC2, which are regulated by PI3K/Akt and AMPK, PPARγ and HIF-1α. These pathways regulate utilization of glycolysis, which promotes pro-inflammatory subset differentiation, or fatty acid oxidation and lipolysis that promote anti-inflammatory Th2 and Treg differentiation. Bottom: Schematic representation how the adipokines leptin (left) and adiponectin (right) promote T helper cell subset polarization *via* regulation of metabolic pathways.

Environmental factors, such as nutrient availability or adipokines, signal molecules secreted by adipocytes, shape not only macrophage function but also the T cell response *via* modulation of metabolic pathways. The most abundant adipokines secreted by the adipose tissue are leptin and adiponectin. While the plasma concentration of leptin, which dampens hunger, increases proportionally with adipose tissue mass and promotes inflammation, adiponectin counters inflammation and supports the Th2 response (**Figure 1**). After we summarize intrinsic regulation of Th2 cell metabolism, we will discuss extrinsic factors that modulate Th2 cell metabolism and hence, function.

### Intrinsic Regulation

#### mTOR

The serine/threonine kinase mechanistic target of rapamycin (mTOR) forms two distinct complexes depending on the scaffolding protein it associates with. The mTOR complex (mTORC) 1 associates with the regulatory-associated protein of mTOR (RAPTOR), while mTORC2 is associated with the rapamycin-insensitive companion of mTOR (RICTOR). The capability to form two distinct complexes allows mTOR to act as a metabolic switch that can exert different functions. mTORC1 integrates primarily signals that indicate favorable conditions for cell growth. In contrast to the sensing of nutrient-availability by mTORC1, mTORC2 can be stimulated by growth factors and cytokines (43). First evidence that mTOR affects T cell differentiation came from studies, which observed that rapamycin treatment inhibited T effector cell differentiation, while allowing expansion of Tregs (44–46). mTOR is a downstream target of PI3K and acts as a crucial global regulator of cellular metabolism (47). PI3K is a kinase activated by factors involved in cell proliferation, such as the epidermal growth factor (48), and cellular metabolism, such as leptin (49). This enables T cells to meet the high energy demand of effector responses following activation (29) and upregulate catabolic pathways, such as glycolysis and lipolysis. mTOR coordinates T cell growth, proliferation, metabolism, and differentiation upon shifts in growth factor and nutrient availability. Activation of mTOR is an important condition for the differentiation of naïve T cells into Th1, Th2 or Th17 cells, while inhibition of mTOR results in primary differentiation into Tregs. Absence of mTOR signaling also leads to a decreased proliferation capacity of T cells (44). Activation of mTORC1 induces lipid synthesis pathways and glycolysis (50) and limits autophagy. mTORC2 regulates cytoskeleton organization in addition to induction of glycolysis (51) and lipolysis. Interestingly, during obesity defective mTOR is observed in NK cells and mucosal-associated invariant T (MAIT) cells: O’Brien and colleagues described an inhibition of mTORC1 in MAIT cells dysregulating their cytokine profile during obesity (18). In contrast, Tobin et al. found an increase of mTORC1 activity in natural killer cells in the adipose tissue of obese children (52). However, these NK cells were reduced in numbers and – despite their increased expression of activation markers – less effective in cell lysis (52). Both, mTORC1 and mTORC2 activate the nuclear hormone receptor PPARγ and are activated by IL-4 (53, 54). Signaling of mTORC1 is enhanced by insulin and influences Th1, Th2 and Th17 cell differentiation (44, 55). Hyperactivation of mTORC1 has been shown to limit invariant natural killer T cells (iNKT) (44, 56). NKT cells are a subset of innate-like T lymphocytes and share characteristics of T and natural killer cells. Once activated, NKT cells can release type 1 or type 2 inflammatory cytokines but the mechanisms of recruitment are not well elucidated yet. The role of NKT cells, which can also be potent producers of IL-4, in the context of immune modulation due to metabolic changes of the individual requires further investigation as these cells are often overlooked (57, 58). Th2 differentiation is more sensitive to graded reductions in mTORC1 activity and can develop in the absence of mTORC1 but not in the absence of mTORC2 (59, 60). While mTORC1 responds to signals including growth factors, oxygen and amino acid availability, mTORC2 primarily reacts to other stimuli. However, it has been shown that signaling of mTORC2 responds to nutrient fluctuations and only promotes Th2 but not Th1 or Th17 cell differentiation (59). These observations lead to the hypothesis that Th2 differentiation is subject to a more dynamic regulation through both nutrients and cytokines/growth factors and is less dependent on nutrient-sensing mTORC1 signaling. Upstream of the mTOR-signaling, activation of the PI3K pathway increases mTORC1 activity *via* AKT, while mTORC2 regulates AKT concentrations and thereby modulates mTORC1 function. Due to the increased nutrient uptake, glucose availability in obesity is increased and subsequently leads to an upregulation of glycolysis and promotion of Th1 differentiation of T cells.

#### AMPK

The AMP activated protein kinase AMPK reacts to the energy level of the cell by sensing the ATP : ADP ratio, regulates glycolysis accordingly and plays a regulating role in energy homeostasis of the cell and T cell activation (61). In a low energy state of the cell, characterized by low ATP levels, AMPK is activated and subsequently inhibits mTORC1 and downregulates glycolytic pathways and Th1 and Th17 inflammation (62, 63). In contrast, AMPK activates mTORC2 and thereby promotes fatty acid catabolism and Th2 and Treg responses (64). In order to generate energy, Th1 cells predominantly upregulate glycolysis, supported by mTORC1 signaling that can directly phosphorylate the Th1 transcription factor Tbet (65), while Th17 cells favor glutaminolysis and regulatory T cells oxidative phosphorylation. The mechanisms of regulation of Treg metabolism are less well elucidated. Compared to naïve T cells, the steady-state activity of mTORC1 is increased and thereby supports the suppressive Treg function partly by inhibiting the mTORC2 pathway in these cells (66). Nevertheless, the predominant metabolic pathways in regulatory T cells are fatty acid oxidation pathways. HIF1α reacts to oxygen stress and decreases Glut1 expression in Th17 cells and regulates c-Myc, a transcription factor that is essential for the metabolic reprogramming early after activation, leading to a decreased activation of the cells. HIF1α signaling is controlled by mTOR. By regulating the Th1 transcription factor Tbet and Eomesodermin (67), a transcription factor amongst others responsible for steering effector T cells into memory T cells, mTOR also determines the T cell fate. Interestingly, Xiong and colleagues found that HIF1α supports Th2 polarization *via* dendritic cells (DC) priming of naïve T cells (68). Taken together, AMPK inhibits type 1 inflammatory responses by inhibiting mTORC1 and supports type 2 inflammatory responses *via* mTORC2.

#### PPARs

The peroxisome proliferator activated receptors (PPAR) are nuclear hormone receptors acting as inducible transcription factors that play a pivotal role in glucose and lipid metabolism. The different isoforms PPARα, PPARβ/δ and PPARγ are primarily activated by fatty acids and predominantly expressed in different tissues, with PPARα being found especially in tissues with increased fatty acid oxidation, such as hepatocytes (69), and PPARβ/δ in the gastrointestinal tract (70). PPARγ is considered the master regulator of adipogenesis and highly expressed by adipocytes as well as macrophages and CD4^+^ T cells within the adipose tissue (71, 72). PPARγ acts as a sensor of the metabolic status of the cell and regulates glucose metabolism and lipid storage, as well as adipocyte differentiation (73). PPARγ is prominently expressed in Th2 cells and has been shown to have an inhibiting effect on Th17 inflammation while strongly supporting Th2 and Treg responses in the adipose tissue (74, 75). Activation of PPARγ by mTOR leads to an increase of fatty acid uptake, increased plasma concentrations of adiponectin (76) and an induction of adipogenesis, reviewed in (77). PPARγ function is supported by the adipokine adiponectin, that is found in higher concentrations in individuals with a lean bodyweight compared to those with obesity. Consequently, a correlation between PPARγ activation and weight loss can be observed (78). Importantly, in mice on HFD, IL-4 promoted lipolysis and weight loss through PPARγ downregulation in adipocytes (79). PPARγ has also been described as an important inducer of regulatory T cell generation (80).

Kopf and coworkers found that PPARγ plays a pro-inflammatory role in type 2 immunity and is an important mediator for DC-T cell interactions (81). While PPARγ is largely dispensable for the induction of IL-4 production, IL-4 and IL-33 promote the up-regulation of PPARγ in lung resident CD11b^+^ DCs, which leads to an enhanced migration to the draining lymph nodes and Th2 priming capacity (81). Micossé et al. defined a phenotypically and functionally distinct Th9 phenotype of T cells that could be a subtype of Th2 cells that are defined by PPARγ expression (82). The cytokines IL-4 and TGFβ both induce PPARγ and leads to IL-9 production in Th2 cells. Meta-analytic approaches identified a possible contribution of PPARγ to a decreased susceptibility for type two diabetes mellitus in different ancestries (83).

Taken together, these results suggest that a metabolic profile characteristic of activated CD4^+^ T cells leads to differentiation into type 1 inflammatory Th1 cells, while the expansion towards Th2 and Treg cells is supported by a metabolic profile characteristic of quiescent cells (**Figure 1**). As type 1 inflammatory responses are mostly directed to respond to acute viral or bacterial infections a rapid cell proliferation is necessary so that the host can react quickly to the pathogen. Consequently, metabolic pathways that can be upregulated quickly, such as glycolysis, are important in this context. In contrast, type 2 inflammatory responses are directed against continuous inflammatory insults, such as helminth parasite infections or exposure to allergens. In such chronic infections or prolonged exposure and allergen sensitization, no rapid response is necessary and mitochondrial pathways, such as the TCA cycle and oxidative phosphorylation, can be utilized. Additionally, oxidative phosphorylation produces reactive oxygen species that may negatively affect a fast T cell response.

### Extrinsic Regulation

Cell metabolism is regulated by various extrinsic factors. Hormones or cytokines influence the metabolic regulation as well as nutrient availability in the cell, such as glucose, by regulating transporter expression in the cell. In obesity, not only the nutritional status of the individual changes, but also concentrations of messenger molecules, such as leptin, adiponectin, resistin, visfatin, and others, which may directly affect Th2 cell function or indirectly inhibit Th2 cells by promoting Th1/Th17-biased inflammation. Adipose tissue produces the hormone leptin and with the expansion of adipose tissue in obesity, the leptin concentration increases. In contrast, the concentration of adiponectin, a hormone regulating the glucose metabolism, decreases following the onset of obesity. Adiponectin is secreted by adipocytes, as well as lymphocytes (84) and has anti-inflammatory properties, such as limiting IFNγ production (85) or promoting IL-10 secretion by Tregs (86). An important regulator of the blood glucose concentration is the hormone insulin, produced by the β cells of the pancreas (87). By inducing glucose uptake into the tissue, insulin is an anabolic hormone whose effect on T cells is only started to be researched in detail. The underlying chronic Th1 inflammation in individuals with obesity leads to an increase of pro-inflammatory cytokines, such as IFNγ, while anti-inflammatory cytokines, such as IL-10 are decreased. IL-33 is an alarmin that has been found to induce type 1 as well as type 2 responses. The cytokine produced by endothelial and epithelial cells has been shown to have an protective effect on obesity (88). Aside from an oversupply of nutrients that leads to obesity, undernutrition also has significant influence on cellular metabolism and function. The influence of the hormones leptin, adiponectin and insulin, the cytokine IL-33, as well as undernutrition and fasting on T cell function will be discussed below.

#### Leptin

The pro-inflammatory adipokine leptin is secreted by adipocytes and regulates energy consumption and conversion by regulating food intake as well as glucose metabolism (89). Leptin can pass the blood brain barrier and upon binding the leptin receptor in the brain induce various signaling cascades affecting food intake and energy balance. Impaired crossing of leptin of the blood brain barrier, dysfunctions in the subsequent pathways or decreased sensitivity of the leptin receptor lead to a decreased leptin signaling in the brain. These are possible reasons for leptin resistance and can subsequently lead to a dysregulation in the energy homeostasis (reviewed in (90)). Additionally, leptin has been shown to promote T cell survival and proliferation (91). Leptin has a strong positive association with obesity, is expressed at higher levels in metabolically unhealthy PWO and is negatively associated with being underweight and malnutrition (92, 93). The anorexigenic hormone promotes energy consumption by improving glucose metabolism, controlling the appetite and improving insulin sensitivity (reviewed in (89)). During obesity, the serum leptin concentration increases, which can result in a leptin resistance. The consequential increase in food intake, impaired nutrient absorption and inhibition of lipid and glucose metabolism (94) can lead to a further aggravation of the obese phenotype. Especially in individuals with obesity, leptin leads to an increased secretion on IFNγ and suppression of Th2. Naïve T cells lack expression of the leptin receptor, but upregulate it upon activation. In activated CD4^+^ T cells, leptin functions as an activator of mTOR (95), upregulates glucose uptake and metabolism and thereby leads to an increased Th1 (96) and Th17 (97) cellular response and a suppression of Treg cells (98). Indeed, another study showed that leptin enhanced Th1 cytokine production, while IL-4 production was decreased (99). Contrarily, Zhang et al. did not find an effect of leptin on Th2 differentiation but a promoting effect on Th2 survival (100). Moreover, Zeng et al. described an enhancing effect of recombinant leptin on ILC2 and Th2 cytokine expression *via* the PI3K-AKT axis (91, 101). In their study, leptin supported Th1 and Th2 proliferation and survival by activating the (JAK2-STAT3, MAPK and) mTOR pathway. Therefore, they propose the supporting function of leptin on distinct T cell subsets is dependent on the skewing conditions, leading to a leptin-dependent enhancement of a type 2 response in the context of allergy. Consequently, the increased serum leptin concentration could support T cell proliferation and the type 1 biased inflammatory environment presents skewing conditions to support a Th1 differentiation of T cells. Due to increased serum leptin levels in allergic rhinitis, they propose a possible connection of allergic rhinitis to obesity. Taken together, leptin affects Th2 cells indirectly by supporting the underlying Th1 inflammation in obesity.

#### Adiponectin

The protein hormone adiponectin regulates glucose and lipid metabolism. Mechanisms for this regulation include support of fatty acid oxidation and inhibition of gluconeogenesis *via* an activation of AMPK (102). Adiponectin possesses anti-inflammatory properties, such as inducing IL-10 secretion in Tregs (86), and is negatively associated with BMI (103). Furthermore, metabolically healthy PWO often show higher levels of adiponectin similar to the levels of lean people (104). Additionally, two studies found a decrease in glycolysis in activated T cells leading to impaired Th1 or Th17 differentiation (85, 105). Research on the direct effect of adiponectin on Th2 cells is still lacking, but Li et al. could demonstrate a positive effect of adiponectin on IL-4 production that in turn can lead to an increased Th2 response (106). Interestingly, women have more adiponectin in their blood than men. Taking the immunomodulatory effects of adiponectin into account, this could lead to a stronger activation of a type 2 immune response in females (107), which could be relevant to sex differences in obesity and allergic disorders (108). Additionally, as adiponectin promotes insulin sensitivity it supports a potential important player in the context of obesity (109). Taken together, adiponectin is strongly linked to type 2 inflammatory responses, in which its anti-inflammatory effect is further increased (86). Ramos-Ramírez and colleagues demonstrated that adiponectin increased the ability of Treg cells to secrete IL-10 and this effect was further increased in a type 2 inflammatory environment (110). Activation of PPARγ, which induces Th2 and Treg responses, leads to increased adiponectin levels in high fat diet (HFD) fed mice, while overexpression of adiponectin also increases the expression of PPARγ (110). In adipose tissue, adiponectin negatively regulates ILC2 function by activation of AMPK and the subsequent suppression of IL-33 signaling (111). Collectively, these data suggest a supporting role of adiponectin for Th2 responses by inhibiting type 1 inflammatory responses and amplifying IL-4 and IL-10 production.

#### Visfatin, Resistin, Apelin

A number of other adipocyte-derived mediators are increased during obesity, such as visfatin (112), resistin (113) and apelin (114). Visfatin leads to activation of T cells and promotes the release of IL-6, IL-1β and TNF but also IL-10 from monocytes leading to CD4^+^ T cell activation (115). Resistin, which confers resistance to the action of insulin, is considered a pro-inflammatory molecule activating NFκB, TNF and IL-6 (113). However, it has also been shown that modulation of DCs by resistin leads to enhanced Treg expansion (116). Taken together, the direct effect of all three mediators on Th2 cells has not yet been studied in detail.

#### Growth Differentiation Factor 15 (GDF15)

GDF15, which has emerged as a putative target for treating obesity (117), is a stress-induced hormone produced by a variety of cells – including adipocytes - in the body. Recently, it was shown that the Th2 cytokines IL-4 and IL-13 induce GDF15 production by adipocytes in a STAT6-dependent manner (118). Whether GDF15 directly affects Th2 cells *via* its receptor GFRAL is currently unclear.

#### Insulin

The anabolic hormone insulin plays an important role in energy storage, glucose uptake and synthesis of glycogen and lipids. After food consumption, the insulin concentration increases to subsequently increase the glucose uptake from the blood stream into the cells *via* Glut4 (87). In obesity, this increase of insulin concentration is abated and the insulin sensitivity decreases, leading to higher blood glucose levels in the individual. Importantly, metabolically healthy PWO show greater insulin sensitivity than metabolically unhealthy PWO, whereas they are more insulin resistant than metabolically health lean persons (21). Upon activation, T cells begin to express the insulin receptor but contrarily to the anabolic function of insulin within tissue, in T cells insulin signaling supports T cell proliferation and effector function (119). Additionally, insulin influences the differentiation of regulatory T cells by inducing PPARγ (80). Li et al. described an insulin dependent pathway that activates HIF1α and subsequently induces PPARγ (80). Additionally, Jeschke and colleagues reported an anti-inflammatory role of insulin after severe trauma by decreasing type 1 and increasing type 2 inflammatory responses (120). As a possible mode of action, they propose an indirect effect of insulin by reducing of the blood glucose concentration and consequently decreasing glycolysis in effector T cells. Due to glycolysis being the prominent pathway for energy generation in type 1 inflammatory cells, this type of immune response is more severely inhibited. Another mode of action of insulin could be the upregulation of Glut1 on T cells and subsequently increasing the glucose uptake into the cells. Following the increased glucose availability, T cells are steered towards an upregulation of glycolysis and Th1 differentiation. By supporting Th1 differentiation of T cells, insulin could indirectly inhibit Th2 differentiation. Obesity and related pathologies such as type 2 diabetes mellitus (T2DM) increase the blood glucose concentration. Here, insulin resistance that characterizes T2DM could skew T cell differentiation to a Th1 phenotype in a graded manner by a gradual increase of blood glucose concentration and subsequently the nutrients for T cells to utilize glycolysis. Additionally, a strong body of literature shows an association of IL-17 signaling with insulin resistance and the development of obesity and T2DM. Nicholas and colleagues recently described a positive association of Th17 responses with T2DM (121), confirming the positive association of increased Th17 responses during obesity shown by Fabbrini et al. (122). IL-17 directly affects adipogenesis and glucose metabolism as well as impairing insulin sensitivity (123, 124). Thus, interfering with Th cell subset polarization may be a novel therapeutic approach to improve T2DM.

#### IL-33

The alarmin IL-33 is a versatile interleukin that can induce Th1 responses as well as Th2 responses. IL-33 supports IFNγ secretion of CD8^+^ T cells and NK cells (125, 126) as well as neutrophil activation (127), thereby supporting characteristic effector functions of a type 1 inflammatory immune response. However, IL-33 administration can also induce marked type 2 inflammation, with eosinophilia, increased production of the interleukins IL-4, IL-5 and IL-13 as well as mucus production and epithelium remodeling (128). Th2 cells, as well as ILC2, Treg cells and M2 macrophages, express the IL-33 receptor ST2 and are activated by IL-33 ligation to produce type 2 inflammatory cytokines (128). These effects are characteristic for inducing type 2 immune responses. Additionally, IL-33 has been shown to be protective in obesity. In obese mice, administration of IL-33 restores immunological and metabolic profiles of adipose tissue and exerts this effect through activation of adipose tissue ILC2. Treatment of obese mice with ω1, a helminth derived RNAse that induces type 2 inflammatory responses, induced a release of IL-33 from adipocytes that subsequently can further support Th2 responses (129). Mahalakõiv et al. also described a protective effect of IL-33, produced by stromal cells, in diet-induced obesity in mice (130). In a study in humans Tang et al. found elevated levels of IL-33 in Chinese adults with a positive correlation between IL-33 and risk factors for metabolic syndrome (131).

#### IL-25, TSLP

In recent years, also other alarmins, such as IL-25 and thymic stromal lymphopoietin (TSLP), have been investigated and, for both, a supporting role on the Th2 response and protective effect against obesity could be shown. While IL-25 mainly protects from obesity by stimulating M2 macrophages and inducing lipolysis (132–134), TSLP acts trough activation of DCs to prime naïve T cells for differentiation into Th2 cells (135). Treatment of mice on HFD with TSLP protects from weight gain and glucose intolerance, and it further induces the loss of white AT mediated by T cells that upregulate sebum secretion (136). Although increased IL-4 production was observed in TSLP-treated mice, the polarization state of the CD4^+^ T cells was not assessed (136). Thus, there could be a critical role for Th2 cell induction through TSLP-activation promoting an unexpected link to skin barrier maintenance and sebum secretion during obesity.

#### Fasting

While obesity is a well-known and acknowledged health burden, research on the effects of undernutrition on immune cell metabolism and function is sparse. Short-term effects of undernutrition can be assessed through fasting-mimicking diets or intermittent fasting, which has a beneficial effect on the host organisms (reviewed in (137)) and hematopoietic stem cells (138), B cells (139), monocytes (140) and memory T cells (141). Collins and colleagues described a collapse of circulating memory T cells after dietary restriction and an accumulation in bone marrow (141). This effect was even further enhanced by glucocorticoids, steroid hormones that support gluconeogenesis. The accumulation of memory T cells in the bone marrow protected the cells during dietary restriction. Due to the decrease in blood glucose levels following a period of fasting, AMPK is activated and subsequently, mTOR is inhibited to reduce the energy consumption of the cells. Consequently, processes such as autophagy are increased, while glucose uptake and glycolysis are decreased (142). Due to the increased utilization of energy conserving pathways, fasting has especially anti-inflammatory effects, while highly energy consuming pro-inflammatory responses are downregulated (139, 143). Consistent with this, Lenehan et al. found a decrease especially of Th1 and Th17 inflammation in wasting AT of tumor-bearing mice, while the Th2 response were maintained (144). Collectively, fasting has a supporting effect on Th2 responses, while the more energy consuming type 1 inflammatory responses are inhibited.

Collectively, studies indicate that Th2 cells are affected by their nutritional environment (**Figure 1**). While it is known that factors such as mTORC1 activation and leptin signaling inhibit Th2 cells, while AMPK and adiponectin signaling activate or promote Th2 cell function, more research is required to pinpoint the evident differences between the T helper cell subsets. Studies, mainly performed in mice, using different models of obesity and starvation as well as disease models highlight how Th2 cells are influenced by the hosts’ metabolic status.

## Th2 Cell Function During Obesity and Malnutrition

**Figure 2:**
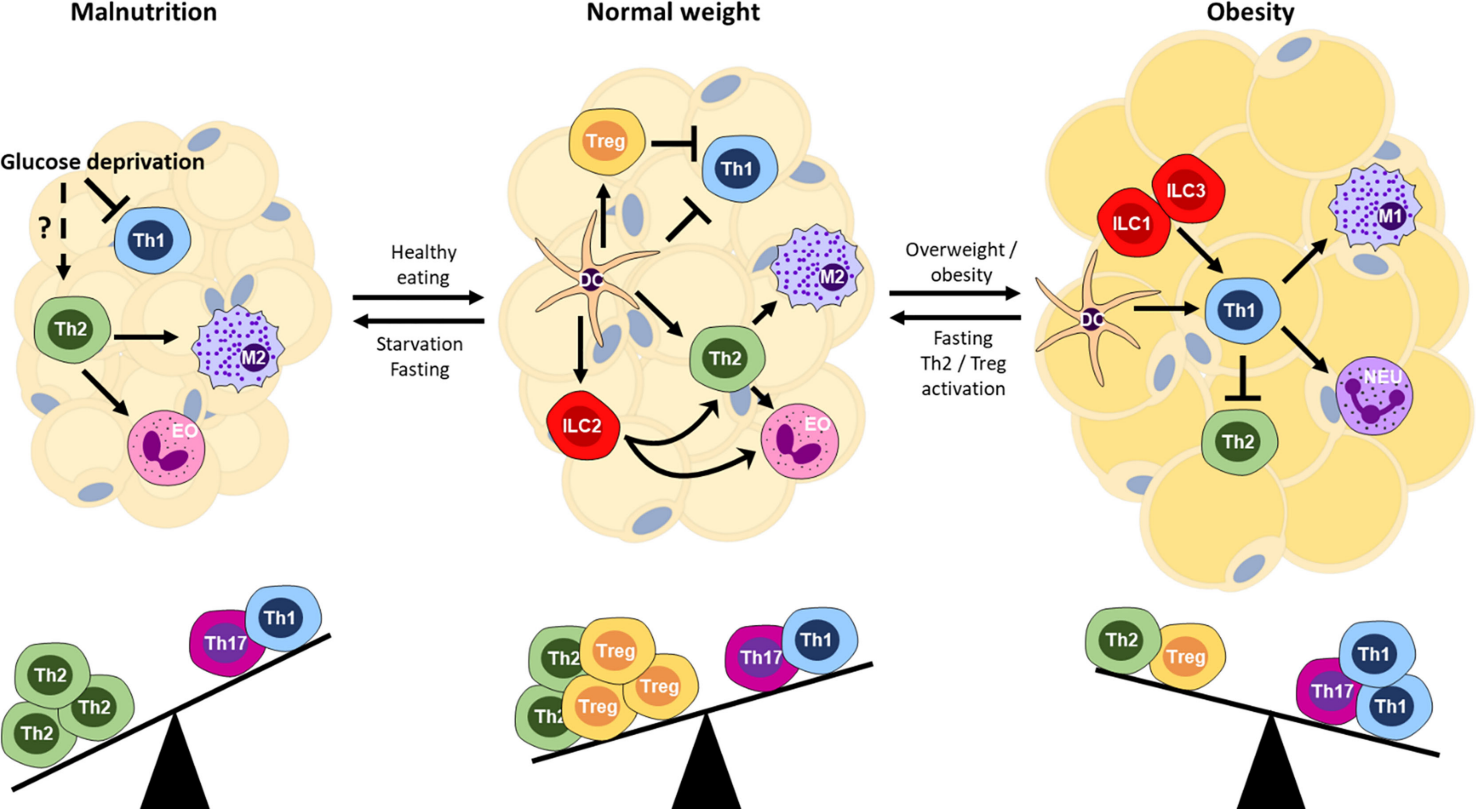
Immune balance and the nutritional state of the host organism. Schematic of cellular interactions within adipose tissue that affect T cell subsets and downstream effector mechanisms. Th, T helper; Treg, regulatory T cells; DC, dendritic cell; M1, M1/classically activated macrophages; M2, M2/alternatively activated macrophages; EO, eosinophil; NEU, neutrophil; ILC1/2/3, innate lymphoid cell type 1/2/3.

**Figure 3:**
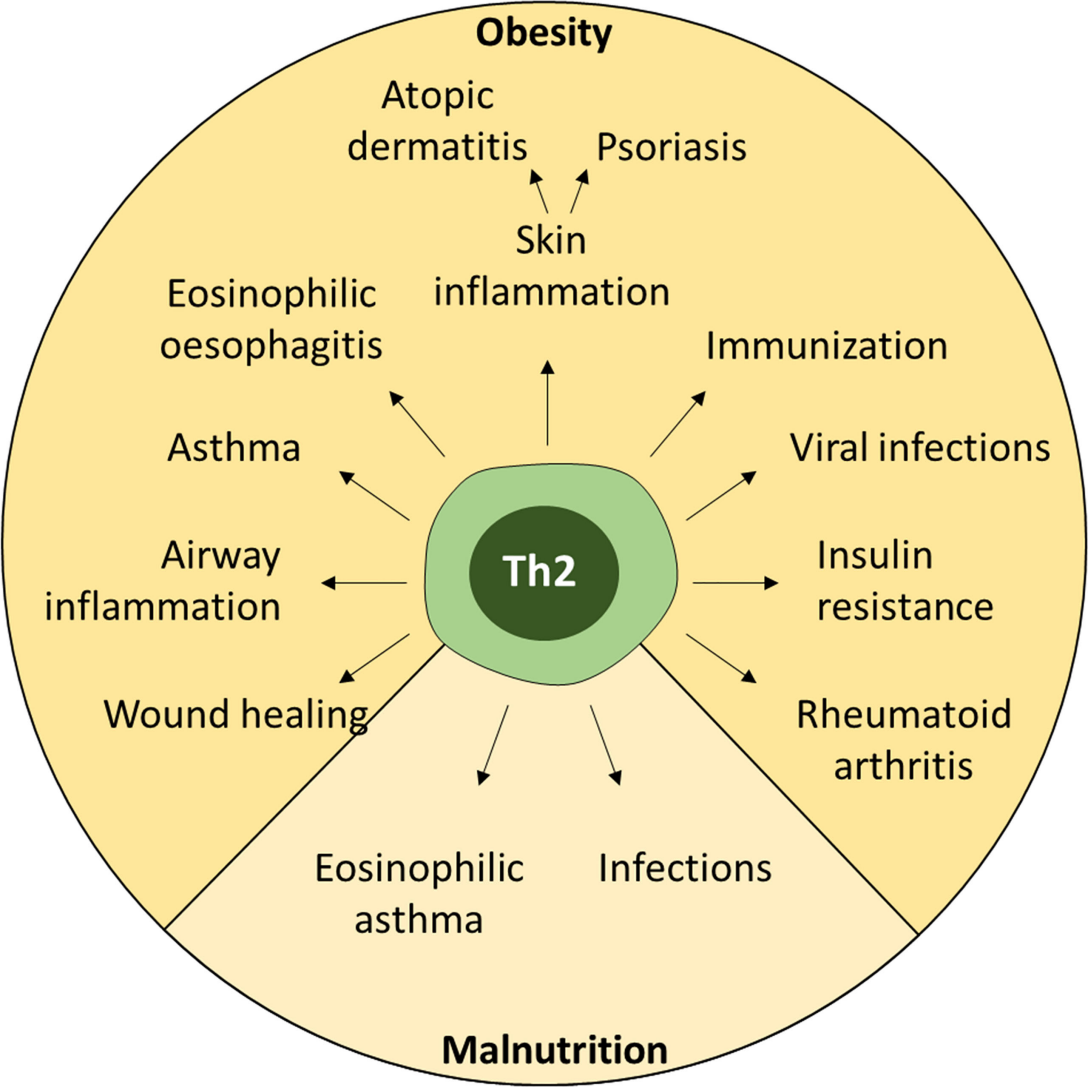
Effects of nutritional dysregulation on peripheral immune responses. Both obesity and malnutrition lead to dysregulation of Th2 cells, which can affect many different inflammatory conditions ranging from allergies to infections and autoimmunity.

The regulation of CD4^+^ T cell metabolism on a cellular level is an important factor for the activation, differentiation and polarization of the cells. The impact of metabolic regulation also affects immune responses both locally within adipose tissue (**Figure 2**) and in the periphery (**Figure 3**). During obesity, T cells live in a nutrient-rich environment, in which fatty acids are abundantly present, whereas during malnutrition glucose availability is decreased. However, due to the systemic immune and hormonal alterations in an obese or fasted state it is difficult to disentangle effects of nutrients and cytokine/hormone activities and this area is only beginning to be studied in detail. Therefore, we will summarize the currently available literature on local – within AT- and peripheral Th2 cell function during obesity and fasting (**Figure 3**).

### Local Effect on Adipose Tissue Th2 Cell Function

Within the healthy adipose tissue, an anti-inflammatory environment predominates. Several cell types that are associated with a classical type 2 response have been identified in the adipose tissue, including ILC2, eosinophils and M2-polarized macrophages (145, 146). However, data concerning Th2 cells in adipose tissue are limited.

During obesity, Th1 cells increase within the adipose tissue, which could have an inhibitory effect on Th2 cells through the production of IFNγ (147, 148). IFNγ induces SOCS1 that inhibits IL-4R signaling (149, 150). Furthermore, the Th1 transcription factor Tbet directly interferes with GATA3 (151). Interestingly, the absolute number of Th2 cells remains relatively constant (152), suggesting that cell proliferation is inhibited. Other mechanisms than the modulators, including leptin and adiponectin, outlined above, may also play important roles in the suppression of Th2 cells during obesity. We have recently shown that PD-L1-upregulation occurs during late stages of obesity in mice and also in human visceral adipose tissue of obese individuals (153), which may block further Th2 proliferation *via* PD-1 engagement. Similarly, regulation of innate effectors, such as ILC2, through cellular interactions shape adaptive T cell responses (154). In essence, obesity disrupts the homeostatic environment in favor of a proinflammatory Th1 bias, the step forwards, while Th2 cells cause a step back from obesity (**Figure 2**).

### Systemic Effect of Obesity on Th2 Cell Function

#### Insulin Resistance

In human subcutaneous and visceral adipose tissue, it was shown that Th2 cells negatively correlate with systemic inflammation and insulin resistance, suggesting Th2 cells have a protective role (25). In mice, transfer of CD4^+^ T cells into obese *Rag1^-/-^* recipients led to the differentiation towards Th2 cells and reversed enhanced weight gain and insulin resistance. These effects were further shown to be dependent on STAT6 (152). In mice on HFD, Th2 cell frequency significantly decreased in the adipose tissue as mice become obese (155). In human adipose tissue, Th2 cells inversely correlated with plasma CRP concentration, a marker indicating systemic inflammation (25). Inflammation has been linked to insulin resistance since the early 1990s, when adipose tissue TNF was shown to be increased during obesity and neutralization of TNF improved peripheral glucose uptake (156). Additionally, IFNγ and IL-1β also modulate insulin signaling (reviewed in (157, 158)), whereas IL-4 and IL-10 were shown to promote insulin sensitivity (159). Given that T cells influence M1/M2-polarization, it is likely that T cell-derived cytokines play an important role for the generation of pro-inflammatory M1-polarized macrophages during obesity (160). Therefore, the decreased production of IL-4 by Th2 cells and IL-10 from Treg cells within the adipose tissue leads to a relative decline of anti-inflammatory M2 macrophages, while the concurrent increase of Th1 cells promotes TNF-expressing M1 macrophages and hence, insulin resistance. Importantly, frequency of Th2 cells in adipose tissue is associated with systemic insulin resistance (152).

#### Asthma

Obesity is associated with an increased risk to develop asthma – a chronic inflammatory disease of the lung. Asthma has a highly heterogeneous pathogenesis and a continuum of endotypes: from a Th2-driven (type 2) to a non-Th2-driven endotype (161). Th2-driven asthma includes early-onset allergic asthma, late onset eosinophilic asthma and exercise-induced asthma, and is characterized by classical type-2 associated factors, including Th2 cells, eosinophils and IgE (162). Non-Th2 asthma includes neutrophilic asthma and obesity-associated asthma, is associated with severe asthma and characterized by Th1, Th17 cells and neutrophils. However, Th2-driven asthma has been described in children and adults with obesity (163, 164) and asthma-obesity endotypes may be associated with age of onset (165).

Dysregulation of T cells may also be an important factor during obesity-induced asthma. Here, the expansion of Th1 cells contributes to the non-Th2-driven asthma pathology. As outlined above, Th2 cells decline with active suppression of Th2 cell differentiation and proliferation by Th1 cell IFNγ and Tbet to counter-regulate Th2 responses. In contrast, it was also found that leptin increased Th2 cells during airway hyperreactivity in mice, in part through MAPK, STAT3 and ER stress response (100, 166). Similarly, in mice on HFD that are immunized and challenged with Ovalbumin (OVA) allergen to induce allergic asthma-like lung inflammation, a mixed inflammatory response developed with increased levels of TNF, IL-5 and IL-10, ultimately leading to an eosinophil-dominated allergic airway response (167). Further studies, have implicated ILC2 and ILC3 (168), or mast cells (169) in asthma pathogenesis, with the latter promoting a delayed Th1, Th2, Th17 profile. A recent study found that autophagy may also be involved in exacerbation of eosinophilic airway inflammation as mice deficient in *Atg5* on HFD had increased Th2 cell numbers in the inflamed lung (170). Interestingly, a 12-week HFD in female mice was protective against airway hyperreactivity through pulmonary DCs recruitment and decreased Th1/17 responses while leaving Th2 cells intact (171). Whereas mice do not develop asthma per se, only aspects of human asthma endotypes can be modelled and should be taken into consideration (161). Taken together, while human obesity-associated asthma is more Th1-driven, in mice – and in certain cases also in humans – a contribution of type 2 associated immune cells including Th2 cells may be important.

#### Skin Inflammation

Inflammatory skin diseases are widespread and have many etiologies. The two most important chronic diseases of the skin are atopic dermatitis (AD) and psoriasis, both of which have been associated with obesity. While AD is classically regarded as a Th2-biased chronic allergic inflammatory skin disease with contribution of eosinophils, basophils, mast cells and ILC2 to pathogenicity, psoriasis is driven by a Th1/Th17-biased immune response and includes neutrophils and ILC3 (172). Both AD and psoriasis have been associated with obesity (reviewed in (173–176)**)**. However, Th2 cells are not directly involved in psoriasis, but a case could be made for diverting the Th1/Th17-response towards a less pathogenic Th2 response by treatment with IL-4 (177).

The overall risk for AD is only minimally increased for adults with obesity (OR=1.08) according to a recent study of patients in the UK (178) but AD is a disease that usually has an early onset during the first years of life. Indeed, several studies have linked childhood obesity to AD (173, 174, 179, 180). However, the pathomechanisms linking these diseases are still not fully understood. Th2 cells seem to be involved, as PPARγ in Th cells drives obesity-associated Th2-immunopathology in severe AD (preprint doi: https://doi.org/10.1101/825836). Furthermore, a recent study in atopic children observed an abnormal blood profile with higher cholesterol and triglycerides (181) – which are also increased during obesity. Whether the increased fatty acids directly influence Th2 polarization *via* PPAR signaling remains unclear (182).

An important factor promoting the development of AD is the breakdown of the skin barrier (172, 183), which can be measured by trans-epidermal water loss (TEWL). Importantly, TEWL is significantly increased in people with obesity (184, 185). The cardinal Th2 cytokines IL-4 and IL-13 may contribute to this breakdown as their homeostatic levels maintain skin barrier integrity (186). Thus, the pro-inflammatory T cell bias during obesity may interfere with homeostatic function or the production of IFNγ, which is also found during chronic AD lesions, may alter skin integrity to disrupt barrier function through alteration of the fatty acid composition of ceramide (187) or downregulation of tight junction function (188). More studies are required to link obesity and the development of a pathogenic Th2 response in the skin.

#### Wound Healing

Obese patients are at risk of developing complications after surgery including wound infections and wound separation (reviewed in (189)), which can be consequences of the impaired wound healing in obese patients (reviewed in Anderson 2015). Th2 cells are an important factor in mediating wound repair by induction of M2 macrophages and eosinophils that promote angiogenesis, myofibroblast activation and deposition of extracellular matrix, as well as by inhibition of pro-inflammatory Th1 and Th17 responses (190–192). Whether obesity directly interferes with the capacity of Th2 cells to promote wound healing is currently under investigation.

#### Rheumatoid Arthritis (RA)

A hallmark of chronic synovial inflammation is the sustained influx of immune cells into the joints. Innate effector cells, including neutrophils and pro-inflammatory macrophages, and effector T and B cells promote synovial inflammation. The anti-inflammatory cytokines IL-4, IL-13, IL-10 and TGFβ are downregulated during established RA (193). However, during early RA IL-4 and IL-13 are upregulated in synovial fluid suggesting an early modulatory or compensatory role, also highlighted by their anti-arthritic properties (194–197). In a mouse model of collagen-induced arthritis, Th2 cells were shown to be increased after repeated IL-33 administration and promoted amelioration of disease (198). People with obesity that additionally have RA, also have poorer quality of life and show higher disease activity (199). To date it is unclear how Th2 may be affected in RA patients with obesity. It is tempting to speculate that the pro-inflammatory state in obese individuals negatively affects RA pathogenesis and disease activity, which is ameliorated through the induction of Th2 cells.

#### Eosinophilic Oesophagitis (EoE)

EoE is an emerging disease, which is characterized by a deregulated type 2 response with high numbers of eosinophils in the oesophageal epithelial layer, leading to clinical symptoms that include dysphagia, feeding dysfunction and vomiting. In a model of OVA-induced EoE, obesity increased Th2 cytokine expression and reduced regulatory T cell responses (200). The altered immune response was not only limited to the oesophageal tissue but expanded to the spleen and mediastinal lymph nodes. Whereas Th2 cells were increased, OVA-specific IgE responses were reduced in obese allergic mice (200). Leptin levels were increased in obese allergic mice, which may affect T cell polarization as outlined above, but more experimentation is required to formally address this in the context of EoE. Whether the exacerbation of Th2 responses during obesity may be involved in other allergic settings, such as food allergies, and how these processes can be therapeutically exploited must be the focus of future studies.

#### Infectious Diseases

Obesity is associated with increased risk to contract infectious diseases including skin infections and infections of the lower respiratory tract (201). Importantly, differences between men and women were also noted. Similarly, obesity affects the outcome of infections (202). During the SARS-CoV-2-pandemic it also became evident that obesity is an important risk factor for severe disease and mortality (203). Remarkably, Th2-associated cytokines showed an upward trend over the severe courses of Covid-19 (204). Similarly, IL-13 was found to be a driver of severe disease (205) promoting lung damage with participation of Th2 cells (206). However, it is currently unclear to what extent Th2 cells and the associated cytokines contribute to pathology in severe Covid-19.

In conclusion, obesity is an important factor regulating Th2 functionality extending beyond local tissue responses. However, to date studies investigating Th2 cells in other inflammatory settings are scarce and such studies may reveal unexpected novel regulatory functions of Th2 cells.

### Systemic Effect of Fasting/Malnutrition on Th2 Cells

Obesity is a consequence of overnutrition and its high prevalence in industrialized countries led to a heightened research interest in this area, whereas the study of the effects of malnutrition on the immune system is less widespread. Immunity in the context of chronic malnutrition is difficult to study and according to a 2014 systematic review yields varying results (207). However, starvation and cachexia are linked to immune dysfunction in humans and mice (208). After two days of starvation, splenic CD4^+^ T cells in mice declined by 50% (209), which can also be observed in malnourished children (210). Importantly, not only numbers of Th cells were reduced in the blood of starved mice and also malnourished children, but their capacity to produce IFNγ and IL-2 (209, 211). Instead, malnourished children had increased concentrations of IL-4 and IL-10 in their blood (212). In the absence of glucose, activation of Th cells *via* Glut1 is impaired (8), but whether this also impairs Th2 cell function, which seems to be intact although glycolysis cannot be used (213), remains to be determined. Interestingly, a recent study showed that during cancer-associated adipose tissue wasting the Th2-environment in adipose tissue is maintained both in mice and humans (144).

Intermittent fasting, which has become a widespread strategy to lose weight, is another approach to investigate the effects of short-term caloric restriction on immune cells. In a mouse model of eosinophilic asthma - induced by intranasal administration of IL-33 and OVA - a two-day fasting period reduced the accumulation of Th2 cells, IL-13, generation of OVA-specific IgG1, and eosinophils in the lungs compared to non-fasted mice (142). Whether this effect is solely attributable to Th2 cells or mediated by ILC2s remains to be determined. During a model of autoimmunity, fasting reduced accumulation of CD4^+^ T cells and IFNγ-producing cells, while it increased IL-17A production (214). Taken together, while Th2 cells, and indeed other T helper subsets, are affected by malnutrition well-controlled studies that address the functional impact on immunity are required.

### Helminth-Mediated Induction of Th2 Cells During Obesity

Taking into account that Th2 cells are beneficial for the homeostatic environment in adipose tissue of lean individuals, it is only logical that the question arises whether induction of Th2 cells during obesity has an ameliorating effect. Several studies have addressed this question using helminth infections to evoke a Th2 and regulatory state that may impact the severity of unrelated inflammatory conditions and disease states. There are extensive studies in mouse models, as well as human clinical trials using live helminth infections for the treatment of several inflammatory diseases (reviewed in (215)).

The impact of the induction of Th2 cells by helminth infection on the low-grade chronic inflammation during obesity has been addressed in a number of studies. Studies in helminth-infected humans observed improved metabolic health (216–223). In mice, acute helminth infection with *Nippostrongylus brasiliensis* also improved metabolic health and decreased weight gain (146, 224). Hussaarts and colleagues revealed that chronic *Schistosoma mansoni* infection (>12 weeks) significantly ameliorated diet-induced obesity in mice compared to non-infected controls (225). Unsurprisingly, infection with the gastrointestinal helminth *Heligmosomoides polygyrus* also led to ameliorated weight gain and improved metabolic function (226). Su et al. brought more complexity to these findings as they show that Th2 cells were critical in the regulation of the helminth-induced changes to the microbiome that subsequently affected nutrient uptake and weight gain (227). Studies with *Strongyloides stercoralis*-infected patients that were obese revealed that they had also higher type 2-associated cytokines in their circulation (228). Interestingly, these changes were reversed upon antihelminthic treatment to remove the worm infection.

While these infection models rely on infection with live helminths, the side effects may outweigh the benefits and thus single helminth-derived products may have a better safety profile for human application. Omega-1, derived from *S. mansoni* eggs, that is a potent inducer of Th2 cells in mice, was shown to ameliorate HFD-induced obesity (129, 229). More recently, ES-62, a glycoprotein of *Acanthocheilonema vitae*, was also shown to improve metabolic health (230). Importantly, small-molecule analogues still possess anti-inflammatory properties (231). With regard to the variety of helminth-derived products and their immunomodulatory functions (reviewed in (232)), many of these may be able to modulate Th2 cell function during obesity and thus may improve diabetes or even counteract weight gain.

In contrast to these hypothetical experimental interventions, there is also potential to target T cell metabolism using 2-deoxyglucose (2-DG), which inhibits glycolysis and blocks CD4^+^ T cell proliferation, with 2-DG used in numerous clinical trials focusing mostly on cancer but to date none have investigated a possible impact on AT T cells and obesity. Leptin has also been used in clinical trials exploring therapeutic potential in obesity (233). It is clear that further insight on the regulation of different T cell subsets in the context of obesity and, or, the metabolic syndrome are required to inform on the development of new T cell mediated therapies for obesity.

## Conclusion

In recent years, the importance of adipose tissue as an immune organ has become increasingly appreciated. It has been shown that changes in the metabolic status of an individual subsequently lead to changes in the immune balance. Overall, obesity primarily impairs Th2 responses, while reinforcing type 1 inflammatory responses. Starvation and malnutrition, on the other hand lead to a type 2 biased immune response. However, more research on the metabolic pathways promoting T cell polarization towards Th2 is still required, especially with regard to the temporal regulation of the utilization of glycolysis and OXPHOS during different immune responses. Further investigation into the importance of IL-4-producing iNKT cells will also help to develop further therapeutic options, such as treatment with αGalCer for the activation of iNKT cells (234), IL-25, TSLP or IL-33 to activate ILC2 and Th2 cells (136, 235–237), or IL-4 or helminth products to promote Th2 differentiation (238). In the ongoing immunometabolism dance, it is one step forward and (Th)2 steps back.

## Author Contributions

VS, AH, PF, and CS wrote the manuscript. PF and CS conceived the manuscript. All authors contributed to the article and approved the submitted version.

## Funding

PF was supported by the National Children’s Research Centre and Science Foundation Ireland (10/IN.1/B3004). CS is supported by the Interdisciplinary Center for Clinical Research (IZKF) at the University Hospital of the University of Erlangen-Nuremberg (J79), the Else Kröner-Fresenius-Stiftung (2019_A181) and the Federal Ministry of Education and Research (BMBF 01KI2109).

## Conflict of Interest

The authors declare that the research was conducted in the absence of any commercial or financial relationships that could be construed as a potential conflict of interest.

## Publisher’s Note

All claims expressed in this article are solely those of the authors and do not necessarily represent those of their affiliated organizations, or those of the publisher, the editors and the reviewers. Any product that may be evaluated in this article, or claim that may be made by its manufacturer, is not guaranteed or endorsed by the publisher.
